# Psychosocial Health Among Young Adults With Kidney Failure: A Longitudinal Follow-up of the SPEAK (Surveying Patients Experiencing Young Adult Kidney Failure) Study

**DOI:** 10.1016/j.xkme.2023.100763

**Published:** 2023-12-01

**Authors:** Mohammed Al-Talib, Fergus J. Caskey, Carol Inward, Yoav Ben-Shlomo, Alexander J. Hamilton

**Affiliations:** 1 Population Health Sciences, University of Bristol, Bristol, United Kingdom; 2 Richard Bright Renal Unit, Southmead Hospital, North Bristol NHS Foundation Trust, Bristol, United Kingdom; 3 Bristol Royal Hospital for Children, University Hospitals Bristol and Weston NHS Foundation Trust, Bristol, United Kingdom; 4 Exeter Kidney Unit, Royal Devon and Exeter Hospital, Exeter, United Kingdom

**Keywords:** Kidney failure, kidney replacement therapy, KRT modality, lifecourse outcomes, psychosocial health, young adults

## Abstract

**Rationale & Objective:**

There have been no longitudinal studies examining the evolution of psychosocial health of young adults with kidney failure as they age. We aimed to address this in the Surveying Patients Experiencing Young Adult Kidney Failure-2 (SPEAK-2) study.

**Study Design:**

5-year follow-up longitudinal survey of the original SPEAK cohort.

**Setting & Participants:**

16- to 30-year-olds in the UK receiving kidney replacement therapy (KRT) between 2015 and 2017 who participated in the SPEAK study.

**Exposure:**

Kidney failure and KRT modality.

**Outcomes:**

Psychosocial health and lifestyle behaviors.

**Analytical Approach:**

Within-cohort changes in psychosocial health were analyzed using the paired *t* test, Wilcoxon signed-rank test and McNemar’s test. We compared responses to the age-matched population and examined the impact of changes in KRT modality on psychological health using linear regression for continuous outcome variables as well as logistic, ordered logistic and multinomial logistic regression for binary, ordered categorical and unordered categorical variables, respectively.

**Results:**

We obtained 158 survey responses; 129 had previously responded to SPEAK. Of these, 90% had a kidney transplant. Compared to the general population, respondents were less likely to be married or have children and were more likely to be living with their parents. Respondents had nearly 15 times greater odds of being unable to work due to health (odds ratio [OR] = 14.41; 95% confidence interval [CI], 8.0-26.01; *P* < 0.001). Respondents had poorer quality of life and mental wellbeing and were more likely to report psychological problems (OR = 5.37; 95% CI, 3.45-8.35; *P* < 0.001). A negative association between remaining on or moving to dialysis and psychosocial health was observed, although this was attenuated when controlling for the psychosocial state in SPEAK.

**Limitations:**

Low response rate resulting in imprecise and potentially biased estimates and impact of COVID-19 pandemic while survey was active on psychosocial health.

**Conclusions:**

Young adults with kidney failure have persistent poorer psychosocial health compared to their healthy peers as they age. Our findings also suggest a potential causal relationship between KRT modality and psychosocial health.

## Plain-Language Summary

The psychosocial impact of kidney failure in young adults is implicated in the observed higher risk of transplant loss and death. The Surveying Patients Experiencing Young Adult Kidney Failure (SPEAK) study investigated the psychosocial health of young adults (16-30 years) in the UK receiving kidney replacement therapy and found they had poorer outcomes than the age-matched general population. In this 5-year follow-up study, we observed that as this group matured, they lagged behind their peers in terms of both lifecourse and psychological outcomes. Dialysis recipients had poorer psychosocial health compared to transplant recipients. This emphasizes the lasting impact of kidney failure on young adults' psychosocial health, particularly for those receiving dialysis, highlighting the need for better mental health support and treatment.

Young adulthood is a sensitive developmental period, and the psychosocial impact of kidney failure in this vulnerable group is implicated in the observed high risk for transplant loss and death. In a United Kingdom Renal Registry (UKRR)-based observational study, nearly 1 in 10 young adults starting kidney replacement therapy (KRT) aged 11-30 years died within 5 years.^1^ While transplantation is the treatment of choice, retrospective analyses in the UK and USA identify young adults as the highest risk age group for transplant loss.^2^^,^^3^

The Surveying People Experiencing Young Adult Kidney Failure (SPEAK) study provided detailed information of the psychosocial impact of kidney failure on young adults receiving KRT.^4^ This cross-sectional survey of 16- to 30-year-olds in the UK receiving KRT between 2015 and 2017 found that this group was less likely to be in relationships or have children, more likely to live with their parents, and unable to work for health reasons compared to the general population. Respondents had worse quality of life (QoL) and mental wellbeing, with twice the likelihood of psychological disturbance. This was despite more positive lifestyle behaviors, with less smoking, alcohol and drug use. Importantly, mental wellbeing and medication adherence were negatively associated with psychological morbidity and dialysis treatment.^5^

While the psychosocial challenges young adults receiving KRT face are increasingly recognized, there have been no longitudinal studies investigating the natural history of these outcomes as young adults age. To address this, we designed SPEAK-2, a 5-year follow up of the original SPEAK study cohort. Our aims were i) to describe how the psychosocial health of young adults with kidney failure changes over time, ii) to compare these psychosocial outcomes with equivalent general population data, and iii) to explore the relationship between psychosocial health and changes in KRT modality.

## Methods

The SPEAK-2 study is a 5-year follow-up longitudinal online survey study of the original SPEAK cohort. SPEAK-2 was granted ethical approval by the Health Research Authority National Research Ethics Service Committee Brent (reference 20/LO/0534). The study was funded by a Bristol Health Research Charity (charity number: 248189) Clinical Research Fellowship.

### Study Participants and Data Collection

SPEAK-2 participants were individuals recruited to the original SPEAK study (hereafter referred to as SPEAK-1) who had consented to be contacted for future studies. Inclusion criteria for SPEAK-1 were (1) 16 years and older and younger than 31 years and (2) receiving long-term KRT. Consent for participation in SPEAK-1 allowed for linkage to UKRR data for baseline demographic and clinical information, regardless of whether the individual responded to the survey.

SPEAK-1 consent forms were screened to exclude individuals not consenting to be contacted for further studies. The UKRR was used to identify and exclude participants who had died since SPEAK-1. Linkage also provided up-to-date address information to facilitate study invitation. Individuals with no contact details available were excluded.

We invited 879 eligible participants via email and/or postal invitation between June 2020 and January 2021. Invitations consisted of a Patient Information Sheet and a quick response (QR) code and hyperlink directed to an e-consent form. On consenting, participants could access the survey. Email reminders were sent after 2 weeks and then monthly to individuals who had either not commenced or had partially completed the survey. Identifiable information (name, date of birth) on consent forms were used to link responses to SPEAK-1.

### Survey Items

Questions were derived from validated health surveys as previously described.^4^ A virtual patient and participant involvement group guided which elements of the original study to retain. Sections on smoking, alcohol, and drug use were shortened given that adverse lifestyle outcomes were uncommon in SPEAK-1. Given that the patient and participant involvement group identified changes to available income support, relevant questions were updated to reflect current provisions. Due to postpublication difficulties with the scale used to measure medication adherence in SPEAK-1, the Medication Adherence Rating Scale (MARS) was used as a substitute in SPEAK-2.^6^ A summary of other scales used is reported previously.^4^

### Survey Software

Study data were collected and managed using Research Electronic Data Capture (REDCap). REDCap is a secure web-based application hosted at the University of Bristol that supports data capture for research studies. REDCap provided greater convenience than a paper survey; reduced printing, postage, and data entry costs; and reduced the risk for data entry errors.

### Statistical Analysis

Scale author recommendations or published and validated methods were used to handle missing data. We used Pearson’s χ^2^ tests to examine demographic differences between SPEAK-1 and SPEAK-2 respondents.

For the internal comparison of how psychosocial health changed over time, we restricted the analysis to individuals who had responded to both SPEAK-1 and SPEAK-2 surveys. We used paired *t* tests and Wilcoxon signed-rank tests for paired continuous parametric and nonparametric data, respectively. We used McNemar’s test for paired binary data.

Psychosocial outcomes were compared against the age-matched general population based on data from the Health Survey for England (HSE) 2012 to assess how changes in psychosocial health compared to the general population. SPEAK-2 responses were weighted as the inverse of the sampling fraction for sex and socioeconomic status to better represent prevalent young adults on KRT and increase generalizability. Weighting was undertaken using summary-level UKRR data for individuals aged 19-35 years receiving KRT in the UK. Outcomes were compared using linear regression for continuous outcome variables and logistic, ordered logistic and multinomial logistic regression for binary, ordered categorical and unordered categorical variables, respectively. Models were adjusted for age and sex. β-Coefficients are reported for continuous measures, and odds ratios (OR) are reported for logistic and ordered logistic models. For multinomial logistic regression models, relative risk is reported.

The analysis of the relationship between change in KRT modality and psychosocial health was performed using regression. Participants were stratified into groups reflecting their KRT modality status between studies: 1) remained with transplant, 2) moved from dialysis to transplant, and 3) moved from transplant to dialysis or remained with dialysis. We developed models adjusted for age, sex, and for the outcome measure of interest at baseline to explore the potential causal relationship between KRT modality and psychosocial health outcomes. For the baseline-adjusted models, participants who had not responded to SPEAK-1 were by necessity excluded from the analysis.

## Results

### Survey Response

Recruitment is summarized in Figure S1. Of the 976 SPEAK-1 participants, 59 did not consent to be contacted for future studies. UKRR linkage identified 22 individuals who had died between studies. Of the remaining 895 eligible participants, 16 were excluded due to lack of contact details, and an additional 6 requested study database removal after invitation. Overall, 879 individuals were invited to participate in SPEAK-2. There were 158 respondents (response rate 18%). Of the respondents, 129 (82%) had also previously responded to the SPEAK-1 survey. There was no association between baseline General Health Questionnaire (GHQ)-12 score and response to SPEAK-2 (*P* = 0.1).

### SPEAK-2 Respondent Characteristics

As shown in Table 1, SPEAK-2 respondents who had also responded to SPEAK-1 were 45% male, 92% white and had a median age of 30.5 years. Most respondents remained with a functioning transplant between SPEAK-1 and SPEAK-2 (70%), while 20% had moved from dialysis to having a kidney transplant. The minority of participants either remained on dialysis between studies (4%) or had moved from transplant to dialysis (6%). Demographic characteristics of SPEAK-2 respondents who did not respond to SPEAK-1 and SPEAK-2 respondents overall are presented in Table S1. Characteristics of SPEAK-1 respondents who did not respond to SPEAK-2 were described previously.^7^

**Table 1:** Demographic and Clinical Characteristics of SPEAK-2 Respondents Who Had Previously Responded to SPEAK-1

Respondent Characteristics	n
	(proportion)
**Sex**	**124**
Male sex	56 (45%)
**Age group**	**129**
<21 y	5 (4%)
21 to <26 y	27 (21%)
26 to <31 y	39 (30%)
≥31 y	58 (45%)
**Ethnicity**	**129**
White	119 (92%)
Asian	5 (4%)
Black	4 (3%)
Other	1 (1%)
**Index of multiple deprivation quintile (1** **=** **least deprived, 5** **=** **most deprived)**	**106**
1	15 (14%)
2	18 (17%)
3	25 (24%)
4	17 (16%)
5	31 (29%)
**Current KRT modality**	**120**
Kidney transplant	108 (90%)
Hemodialysis	11 (9%)
Peritoneal dialysis	1 (1%)
**Change in KRT modality between studies**	**116**
Remained with kidney transplant	81 (70%)
Remained on dialysis	5 (4%)
Moved from dialysis to kidney transplant	23 (20%)
Moved from kidney transplant to dialysis	7 (6%)

*Note*: Total n=129. Participant interaction with the online survey led to the generation of a unique identifiable record and counted as a response. Percentages may not total 100 due to rounding. N reported for each characteristic represents the number of respondents for whom data was available from both SPEAK-1 and SPEAK-2.

Abbreviation: KRT, kidney replacement therapy.

### Psychosocial Health Changes Over Time

#### Lifecourse outcomes

Paired analyses were conducted considering respondents to each survey item in both studies (n=129; Table S2). In SPEAK-2, more participants were married or in a civil partnership (17% vs 9%, *P* = 0.02), able to drive a car (77% vs 68%, *P* = 0.002), and had a university-level degree or higher-level education (57% vs 44%, *P* = 0.002). They were less likely to be living with their parents (40% versus 63%, *P* < 0.001). No differences in terms of homeownership were observed.

#### Psychological outcomes

Self-reported psychological outcomes are presented in Table 2, with additional scales presented in Table S3. Using a GHQ-12 cutoff of ≥4/12 to define probable psychological disturbance or mental ill health, a greater proportion of SPEAK-2 participants had evidence of psychological morbidity (45% vs 24%; *P* < 0.001). They had inferior mental wellbeing (Warwick-Edinburgh Mental Wellbeing Scale [WEMWBS]; β = -1.76; 95% CI, -3.27 to -0.25; *P* = 0.02). No differences were identified in domains including QoL (EQ-5D-3L), independence with activities of daily living (IADL), body image, perceived social support, and acceptance of illness.

**Table 2:** Changes in Paired Self-Reported Psychologic Health Outcomes Among SPEAK-1 and SPEAK-2 Respondents

Psychological Outcome	n	Possible Range	SPEAK-1		SPEAK-2		*P*
			Median (IQR)	Proportion (n)	Median (IQR)	Proportion (n)	
EQ-5D-3L tariff	114	-0.59 to 1.00	0.85 (0.69-1)		0.85 (0.69-1)		0.62
"No problems" across all EQ-5D-3L domains	114			44 (39%)		40 (35%)	0.58
Independence with activities of daily living scale	113	9 to 27	27 (24-27)		27 (24-27)		0.06
Fully independent with ADLs (score 27/27)	113			58 (51%)		67 (59%)	0.14
GHQ-12	112	0 to 12	3 (0-6)		1 (0-3)		<0.001
GHQ-12 score ≥4	112			27 (24%)		50 (45%)	<0.001

Psychological Outcome	n	Possible Range	SPEAK-1 (Mean (SD))	SPEAK-2 (Mean (SD))	*P*
WEMWBS Scale	113	14 to 70	48.7 (11.6)	46.9 (10.9)	0.02

*Note*: Total n=129. Nonparametric data are presented as median and IQR. Parametric data are presented as median and IQR. GHQ-12 score ≥4 suggests probable psychological disturbance or mental illness. Lower WEMWBS indicates worse outcome.

Abbreviations: ADLs, activities of daily living; GHQ, General Health Questionnaire; IQR, interquartile range; SD, standard deviation.

#### Comparison to age-matched general population

Respondents remained less likely to be married or in a civil partnership (OR = 0.36; 95% CI, 0.20 to 0.62; *P* < 0.001) and have their own children (OR = 0.21; 95% CI, 0.11-0.37; *P* < 0.001) compared to the general population (Table 3). They were more likely to live with their parents (OR = 3.95; 95% CI, 2.48-6.28; *P* < 0.001). Respondents were almost 15 times more likely to report being unable to work due to health (*P* < 0.001). Those employed were less likely to be working in skilled trades (OR = 0.06; 95% CI, 0.008-0.48, *P* = 0.008). Respondents had similar likelihood of having a university degree or higher education (OR = 1.22; 95% CI, 0.82-1.82, *P* = 0.32) and having ever had sex (OR = 1.84; 95% CI, 0.82-4.13; *P* = 0.14).

**Table 3:** Self-Reported Socioeconomic, Psychological, and Physical Outcomes in SPEAK-2 Respondents and Age- and Sex-Adjusted Regression Analyses Comparing to the Age-Matched General Population

Outcome	n	Weighted Proportion	OR/β (95% CI)	*P*
**Household and employment**				
Married or in civil partnership	122	16%	0.36 (0.2 to 0.62)	<0.001
Living with partner	122	45%	0.54 (0.35 to 0.83)	0.01
Have own children	117	14%	0.21 (0.11 to 0.37)	<0.001
Living with parents	123	44%	3.95 (2.48 to 6.28)	<0.001
Working part-time	107	34%	3.89 (2.22 to 6.83)	<0.001
**Household accommodation if not living with parents**	71			
Renting		54%	1.00 (reference)	-
Own outright		5%	2.01 (0.54 to 7.53)	0.3
Mortgage		35%	0.96 (0.54 to 1.69)	0.88
Rent free		5%	5.98 (1.39 to 25.64)	0.02
**Employment status**	122			
Employed		64%	1.00 (reference)	-
Full-time education		7%	0.98 (0.44 to 2.18)	0.95
Unemployed		5%	1.04 (0.42 to 2.56)	0.94
Unable to work due to health		22%	14.41 (7.97 to 26.05)	<0.001
Homemaker		3%	0.4 (0.12 to 1.37)	0.14
**Job category**	85			
Elementary occupations		12%	1.00 (reference)	-
Managers, directors, senior		6%	1.39 (0.4 to 4.85)	0.61
Professional occupations		17%	2.17 (0.8 to 5.92)	0.13
Associate professional and technical		18%	1.53 (0.54 to 4.34)	0.42
Administrative and secretarial		12%	4.34 (1.44 to 13.09)	0.01
Skilled trades		3%	0.06 (0.01 to 0.48)	0.01
Caring, leisure, and other services		12%	7.18 (2.55 to 20.2)	<0.001
Sales and customer service		13%	3.61 (1.24 to 10.47)	0.02
Process plant and machine operating		6%	0.55 (0.1 to 3.15)	0.5
University degree or higher-level education	121	52%	1.22 (0.82 to 1.82)	0.32
**Age of finishing education**	122			
16 y or under		12%	1.00 (reference)	-
17-18 y		33%	2.66 (1.31 to 5.37)	0.01
19 y or over		48%	2.04 (1.05 to 3.97)	0.04
Not yet finished		7%	1.9 (0.75 to 4.86)	0.18
**Psychological and physical outcomes**				
EQ-5D-3L (no problems across all 5 domains)	119	32%	0.17 (0.11 to 0.26)	<0.001
WEMWBS score	117	46.2 ± 2.17	-6.26 (-8.46 to -4.07)	<0.001
GHQ-12 scale score ≥4	117	44%	5.37 (3.45 to 8.35)	<0.001
Self-reported height (meters)	111	1.69 ± 0.02	-0.04 (-0.06 to -0.02)	<0.001
Self-reported weight (kg)	111	73.9 ± 4.4	-1.03 (-5.33 to 3.27)	0.64
**Lifestyle**				
Ever had sex	96			
Opposite sex only		80%	1.00 (reference)	-
Never had sex		12%	1.84 (0.82 to 4.13)	0.14
Same sex only		2%	1.58 (0.34 to 7.27)	0.56
With both men and women		6%	1.59 (0.55 to 4.57)	0.39
Ever smoked	100	40%	0.61 (0.39 to 0.96)	0.03

*Note*: Data are proportions weighted by sex and IMD to be representative of UK young adults ages 19-35 years receiving KRT. Outcomes are binary unless stated. WEMWBS, height and weight presented as mean ± standard deviation. EQ-5D-3L grouped as “No problems”/“Some problems” in regression analyses, corresponding to tariff of 1 or <1. GHQ-12 scale score ≥4 suggests corresponds with psychological disturbance or mental illness.

Abbreviations: CI, confidence interval; GHQ-12, General Health Questionnaire; OR, odds ratio; WEMWBS, Warwick-Edinburgh Mental Wellbeing Scale.

Respondents had poorer QoL (OR for “no problems” across all 5, EQ-5D-3L domains, 0.17; 95% CI, 0.11-0.26, *P* < 0.001) and poorer mental wellbeing (WEMWBS β = -6.26; 95% CI, -8.46 to -4.07; *P* < 0.001) compared to the general population. They had 5-fold greater odds of psychological problems or mental ill health as measured by GHQ-12 (OR = 5.37; 95% CI, 3.45-8.35; *P* < 0.001).

**Figure 1:**
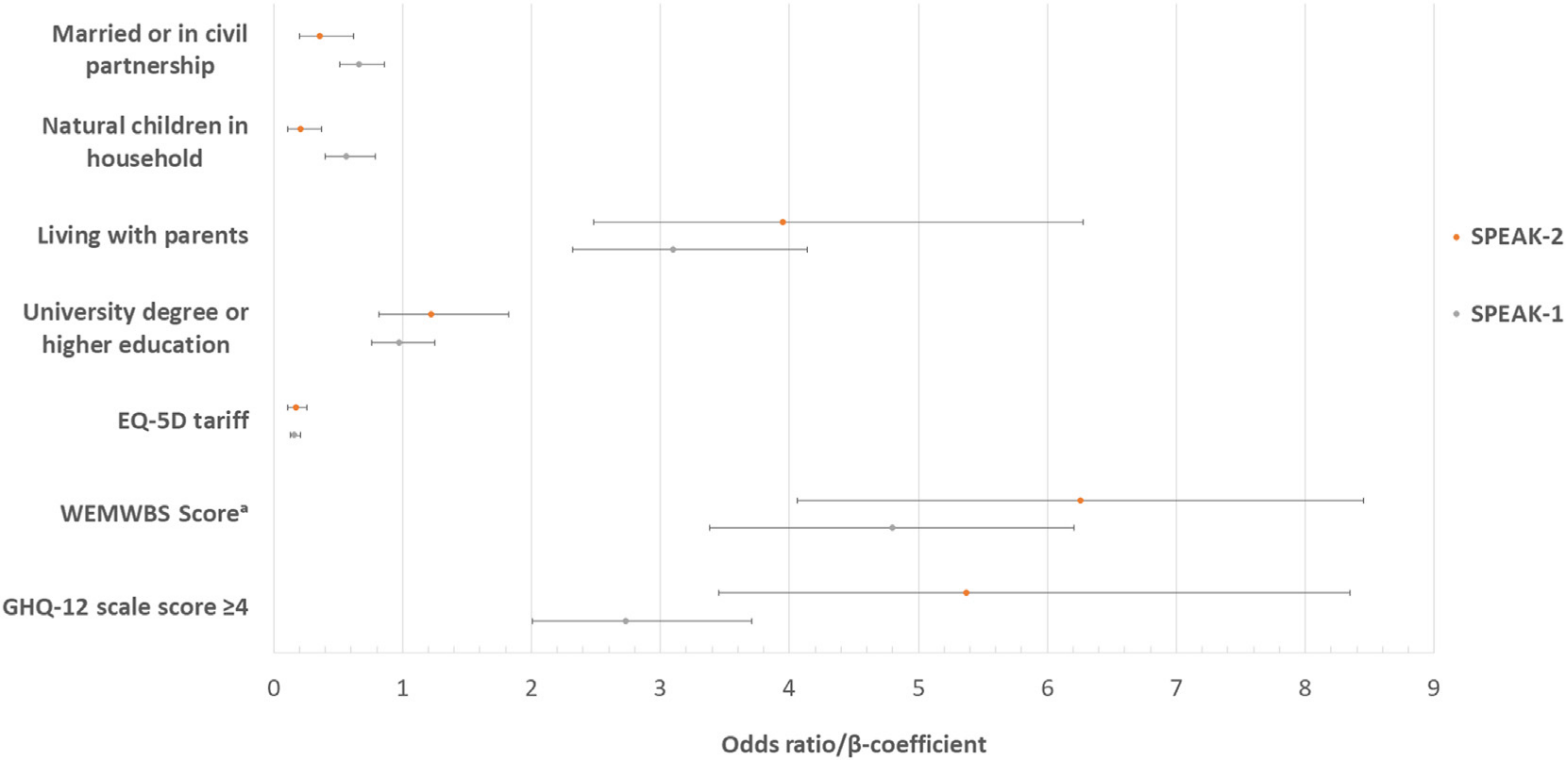
Coefficient plot for age- and sex-adjusted regression analyses comparing selected psychosocial outcomes among young adults receiving KRT against the age-matched general population in SPEAK-1 and SPEAK-2. Outcomes are presented as odds ratio and 95% confidence interval unless specified. Abbreviations: ^a^WEMWBS scores are presented as median and interquartile range and have been transformed from negative to positive values for ease of visualization. WEMWBS, Warwick-Edinburgh Mental Wellbeing Scale; GHQ-12, General Health Questionnaire. GHQ-12 scale score ≥4 suggests corresponds with psychological disturbance or mental ill health.

A comparison of psychosocial outcomes among respondents of SPEAK-1 and SPEAK-2 compared to the age-matched general population is presented in Figure 1. Despite relative improvements in some areas, outcomes compared to the general population were largely similar in both studies suggestive ongoing disadvantage. There were almost double the odds of psychological morbidity (OR = 5.37; 95% CI, 3.45-8.35 vs OR = 2.73; 95% CI, 2.01- 3.71) in SPEAK-2; however, the confidence intervals overlap.

#### Impact of change in KRT modality on psychosocial health

Due to the small number of total respondents that remained on dialysis (n=9, 6%) or moved from transplant to dialysis (n=8, 6%), responses were merged to produce a 3-level exposure for analyses: 1) remained with transplant (n=99, 70%); 2) moved from dialysis to transplant (n=26, 18%); and 3) remained with dialysis or moved from transplant to dialysis (n=17, 12%).

Receiving dialysis was associated with worse psychosocial health across nearly all domains measured (Table 4). We observed a duration effect across most domains, whereby participants who moved from dialysis to transplant had worse psychosocial health than those who remained with transplant. However, their health was not as low as those who remained on dialysis. The exception was body image, where scores of those who had moved from dialysis to transplant were similar to those who remained with transplant (β = -0.34; 95% CI, -3.06 to 2.37; *P* = 0.80).

**Table 4:** Regression Analysis of Psychological Health Scales of SPEAK-2 Respondents by KRT Modality Change Between Studies, Comparing Those Who Moved From Dialysis to Transplant and Those Who Remained on Dialysis/Moved From Transplant to Dialysis to Those Remained With Transplant (Reference)

Psychological Outcome	Adjusted for Age and Sex				Adjusted for Age, Sex and Psychological Outcome in SPEAK-1			
	n	β/OR	95% CI	*P*	n	β/OR	95% CI	*P*
**IADL (fully independent)**								
Dialysis to transplant	142	0.35	0.13 to 0.89	0.03	110	0.44	0.14 to 1.40	0.16
Remained on dialysis/transplant to dialysis	142	0.05	0.01 to 0.27	<0.001	110	0.06	0.01 to 0.57	0.02
**EQ-5D-3L tariff**								
Dialysis to transplant	139	-0.09	-0.22 to 0.03	0.15	111	-0.05	-0.15 to 0.05	0.35
Remained on dialysis/transplant to dialysis	139	-0.43	-0.59 to -0.27	<0.001	111	-0.11	-0.25 to 0.03	0.13
**GHQ-12 score** **≥4/12**								
Dialysis to transplant	137	2.38	0.95 to 5.97	0.06	109	2.75	1.01 to 7.52	0.05
Remained on dialysis/transplant to dialysis	137	4.62	1.33 to 16.09	0.016	109	3.56	0.78 to 16.30	0.10
**WEMWBS**								
Dialysis to transplant	138	-3.69	-8.30 to 0.91	0.12	110	-1.40	-4.86 to 2.05	0.42
Remained on dialysis/transplant to dialysis	138	-9.50	-15.44 to -3.55	0.002	110	-2.68	-7.70 to 2.33	0.29
**BIS**								
Dialysis to transplant	131	-0.34	-3.06 to 2.37	0.80	104	-1.41	-4.37 to 1.54	0.35
Remained on dialysis/transplant to dialysis	131	5.33	1.89 to 8.87	0.003	104	2.19	-1.98 to 6.36	0.30
**SIS**								
Dialysis to transplant	119	6.34	-0.53 to 13.21	0.07	89	0.45	-5.72 to 6.61	0.89
Remained on dialysis/transplant to dialysis	119	15.49	6.71 to 24.27	0.001	89	3.34	-5.31 to 11.98	0.45
**MSPSS**								
Dialysis to transplant	129	-6.57	-12.85 to -0.28	0.04	104	-4.48	-10.74 to 1.79	0.16
Remained on dialysis/transplant to dialysis	129	-3.56	-11.40 to 4.27	0.37	104	2.03	-6.75 to 10.80	0.65
**AIS Scale**								
Dialysis to transplant	128	-4.27	-7.57 to -0.97	0.01	102	-0.88	-4.25 to 2.50	0.61
Remained on dialysis/transplant to dialysis	128	-7.33	-11.37 to -3.29	<0.001	102	-3.33	-7.76 to 1.10	0.14
**PAM-13**								
Dialysis to transplant	115	-6.27	-14.05 to 1.52	0.11	92	-4.91	-12.59 to 2.77	0.21
Remained on dialysis/transplant to dialysis	115	-5.33	-14.95 to 4.29	0.28	92	3.86	-7.11 to 14.83	0.49

*Note:* IADL score of 27/27 indicates “fully independent”. β Coefficients represent the change in scale units and are reported for EQ-5D-3L, WEMWBS, BIS, SIS, MSPSS, AIS, and PAM-13 scales. Odds ratios are presented for other scales.

Abbreviations: AIS, Acceptance of Illness Scale; BIS, Body Image Scale; CI, confidence interval; EQ-5D-3L, EuroQol-5D-3L; GHQ-12, General Health Questionnaire; IADL, Independence with Activities of Daily Living Scale; Multidimensional Scale of Perceived Social Support, MSPSS; Patient Activation Measure-13, PAM-13; OR, odds ratio; Social Impact Scale, SIS; WEMWBS, Warwick-Edinburgh Mental Wellbeing Scale.

When adjusting for baseline scale responses in SPEAK-1 study, the associations observed were attenuated. While point estimates suggested residual poorer psychosocial health, confidence intervals across most domains crossed the null value of 1. The exception was with IADL, for which remaining on dialysis/moving from transplant to dialysis was associated with lower odds of being fully independent (OR = 0.06; 95% CI, 0.01-0.57; *P* = 0.02).

## Discussion

Despite progress in many lifecourse outcomes, young adults receiving KRT continued to lag behind their healthy peers as they age and may transition from youth services to general adult clinics. They remained less likely to be in a relationship or have children and were more likely to be living with parents. Strikingly, they were almost 15-fold less likely to be able to work due to health and reported poor psychological health and lower mental wellbeing, particularly among those receiving dialysis.

The observed progress in lifecourse outcomes since SPEAK-1 ameliorates some concerns raised by young adults with kidney failure. In one qualitative study, they expressed worry about achieving goals, such as finding a partner, having children, and employment prospects.^8^ Internationally, young adults with kidney failure have been shown to achieve comparable educational attainment to the general population; however, this has not translated to equivalent rates of employment.^9^, ^10^, ^11^ While this lifecourse progress observed among our cohort is noteworthy, we recently reported that this was coupled with deterioration in psychological health characterized by 40% of SPEAK-2 respondents reporting symptoms consistent with at least moderate depression and 35% reporting symptoms of at least moderate generalized anxiety disorder.^7^

Compared to the age-matched general population, we observed this cohort continued to lag behind their peers in terms of lifecourse outcomes and psychosocial health. The approximately 15-fold greater odds of being unable to work due to health, similar to SPEAK-1 findings, suggests persistent employment disadvantage. Considering psychological health, the 5-fold greater odds of psychological morbidity per the GHQ-12 scale is particularly striking. This value is almost double what was reported in SPEAK-1, although the confidence intervals overlapped.^4^ The reasons for these sustained poorer outcomes are unclear but do not appear to be explained by deteriorations in body image, stigma, social support, or perception of health care. One explanation could be a long-lasting negative legacy of kidney failure in young adults on life participation. In a thematic synthesis of qualitative studies, the lived experiences of young adults with kidney failure included themes of (1) changes in physical appearance/body image, (2) barriers to activity and participation, (3) educational disruption and underachievement, (4) moderated career ambitions and employment difficulties, and (5) social isolation and intimacy issues.^12^ This was supported more recently by a multinational interview study of young adults with childhood onset kidney disease that highlighted how lifestyle limitations could result in lack of confidence, uncertainty, and vulnerability.^13^ Physical appearance may be difficult to modify with a ‘yo-yo’ effect of alternating modalities of KRT on the body adversely affecting social relationships, as described in one qualitative report.^14^ Similarly, while educational attainment is comparable to the general population, respondents were less likely to be employed in skilled trades, suggesting barriers to employment in sectors requiring dedicated training or perhaps those that are physically demanding. These factors could drive persistent decreased social participation, isolation, and intimacy issues, in turn perpetuating psychological morbidity. This could be explored in future qualitative studies of older adults who developed kidney failure in childhood or young adulthood.

The extent that the COVID-19 pandemic contributed to psychological morbidity is unclear. The World Health Organization reported a 25% increase in anxiety and depression worldwide through to mid-2021.^15^ Likewise, longitudinal surveys of psychological health in UK households demonstrated a general decline early during the pandemic, but this had largely returned to prepandemic levels by the time our survey was active.^16^^,^^17^ However, UK individuals receiving KRT were subject to advice to ‘shield’ until April 2021, and the impact of this on psychological health is uncertain. Mixed methods and cross-sectional studies in other high-risk groups have described a subjective negative impact of shielding on mental health and wellbeing.^18^, ^19^, ^20^ It is uncertain whether further contemporaneous studies examining the psychosocial impact of shielding, in particular on young adults, will emerge.

We found that remaining on or moving to dialysis was associated with worse psychosocial health outcomes compared to remaining with a transplant. This echoes SPEAK-1, where dialysis treatment was associated with poorer mental wellbeing.^5^ Likewise, we recently reported that, among this cohort, depressive and anxiety symptomology was most prevalent among those receiving dialysis.^7^ We observed a duration effect of modality, whereby psychosocial health among those who moved from dialysis to transplant was largely better than those receiving dialysis, but fell below those who remained with a transplant. The exception of body image may reflect a positive effect of dialysis access removal after transplantation. Controlling for the psychosocial state in SPEAK-1 attenuated the relationship between KRT modality and psychosocial health. The low response rate meant confidence intervals largely crossed 1; however, the persistent poorer point estimates observed suggest modality may causally impact psychosocial health. This would support findings from a recent longitudinal cohort study among 377 children in New Zealand that reported improvement in the trajectory of health-related QoL among children receiving dialysis at baseline that was most likely driven by the transition from dialysis to transplantation.^21^ Notably, SPEAK-2 respondents receiving dialysis were significantly less likely to be fully independent (OR, 0.06; *P* = 0.02), even after controlling for IADL in SPEAK-1. This finding is potentially explained by accruing and compounding comorbid conditions among those receiving dialysis.^22^ Indeed, a multinational prospective cohort study demonstrated a high burden of functional dependence among dialysis recipients, and this was a strong predictor of mortality.^23^ We propose that kidney failure early in life has a long-lasting negative psychosocial legacy that is most pronounced in those receiving dialysis. Our findings should strengthen efforts to support young adults to receive a transplant at the earliest opportunity, to engage with treatment and measures to preserve their transplant, and to identify impending graft loss early to plan for pre-emptive retransplantation where possible.^24^

Our study provides the first longitudinal evaluation of psychosocial health of young adults as they age, utilizing validated scales to report outcomes and make general population comparisons. Codesign with patient and public involvement ensured relevant questions were asked. UKRR aggregate data allowed us to weight survey responses, reducing response bias in the general population comparison and increasing generalizability.

Our study has several limitations. Our response rate was low, likely due to our study relying on email and postal invitations, unlike SPEAK-1, which utilized a research network to recruit participants. There was evidence of systematic differences when comparing to respondents of SPEAK-1 that may have introduced bias; however, we observed no association with baseline psychological health and being a responder to SPEAK-2. The small number of participants receiving dialysis necessitated combining groups for analyses. The resultant estimates were imprecise and should be interpreted with caution. Although we controlled for the psychosocial state in SPEAK-1, the observed associations between KRT modality and psychosocial outcomes may be explained by reverse causation. COVID-19 may have contributed to the poorer psychological outcomes observed, and HSE data were collected before the pandemic. We were unable to examine the important association between medication adherence and KRT modality due to the necessary use of a different scale.

In conclusion, in the first longitudinal study examining the psychosocial health of young adults with kidney failure in the UK as they mature, we describe progress in lifecourse outcomes. In particular, educational achievement and entry to professional and managerial roles was equivalent to the age-matched population, making the case for targeted support for skills attainment and workplace entry in social policy. However, respondents trailed the age-matched general population in several areas with worse psychological health over time and striking differences in mental wellbeing, QoL, and psychological morbidity. Our findings suggest that the KRT modality may causally impact psychosocial health, highlighting the need for early intervention to limit the damaging impact of prolonged dialysis treatment.

It is challenging to place these findings in the context of other observational studies examining psychosocial outcomes among young adults as the literature largely focuses on children and transition to adult services; thus, those receiving dialysis or presenting as young adults are underrepresented.^25^, ^26^, ^27^ Future longitudinal follow-up will clarify the extent to which kidney failure in young adulthood impacts psychosocial health in the long term and the impact of interventions.
