# Supplementary material for: Psychosocial Health Among Young Adults With Kidney Failure: A Longitudinal Follow-up of the SPEAK (Surveying Patients Experiencing Young Adult Kidney Failure) Study
Source: Kidney Med. 2023 Dec 1;6(2):100763. doi: 10.1016/j.xkme.2023.100763 (PMC10840100; doi:10.1016/j.xkme.2023.100763)
Supplement: Supplementary File (PDF) — Figure S1, Tables S1-S3. [file mmc1.pdf]

Figure S1: flow chart of recruitment to SPEAK-2 study

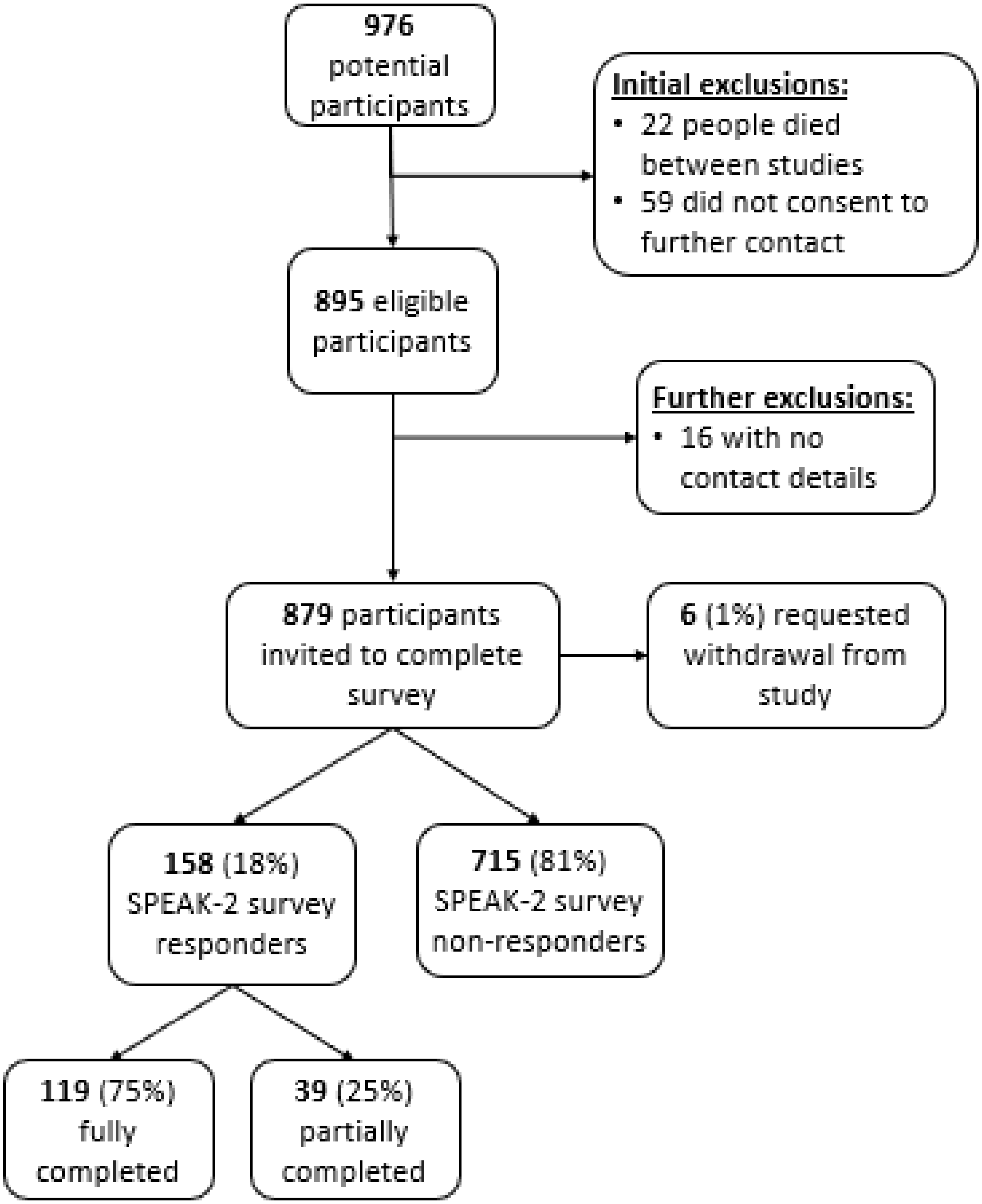

Supplementary figure 1: flow chat of recruitment to SPEAK-2. Note potential 976 participants comprised 625 (64%) individuals who completed the SPEAK-1 survey and 351 individuals who consented to participation in SPEAK-1 study but did not respond to the survey (36%). As such, some SPEAK-2 respondents may not have previously responded to the SPEAK-1 survey. Participant interaction with the

online survey led to the generation of a unique identifiable record and counted as a response. Failure to reach the end of the survey was classed as partial response: what responses provided were included in the analysis.

Table S1: Demographic and clinical characteristics of all SPEAK-2 respondents, and of SPEAK-2 respondents who had not participated in SPEAK-1

**Respondent characteristics**	**All respondents**<br>n (proportion)	**SPEAK-1 non-participants**<br>n (proportion)
**Sex**	**152**	**27**
Male sex	70 (46%)	13 (48%)
**Age band**	**157**	**28**
<21	6 (4%)	1 (4%)
21 - <26	29 (18%)	2 (7%)
26 - <31	54 (34%)	15 (54%)
≥31	68 (43%)	10 (36%)
**Ethnicity**	**157**	**28**
Asian	6 (4%)	26 (93%)
Black	4 (3%)	1 (4%)
White	145 (92%)	-
Other	2 (1%)	1 (4%)
**IMD quintile (1=least deprived, 5=most deprived)**	**130**	**24**
1	21 (16%)	6 (25%)
2	21 (16%)	3 (12.5%)
3	31 (24%)	6 (25%)
4	21 (16%)	4 (17%)
5	36 (28%)	5 (21%)
**Current KRT modality**	**148**	**27**
Haemodialysis	16 (11%)	5 (19%)
Kidney transplant	131 (89%)	22 (81%)
Peritoneal Dialysis	1 (1%)	-
**Change in KRT modality between studies**	**142**	
Remained with kidney transplant	99 (70%)	-
Remained on dialysis	9 (6%)	-
Moved from dialysis to kidney transplant	26 (18%)	-
Moved from kidney transplant to dialysis	8 (6%)	-

KRT, Kidney Replacement Therapy; IMD, Index of multiple deprivation. Participant interaction with the online survey led to the generation of a unique identifiable record and counted as a response. Percentages may not total 100 due to rounding.

Table S2: change in life course and employment characteristics between SPEAK-1 and SPEAK-2

Outcome	n	SPEAK -2	SPEAK-1	*P*
**IMD Quintile: 1[least deprived],…, 5[most deprived]**	106	14%, 17%, 24%, 16%, 29%	25%, 21%, 21%, 12%, 21%	0.14
**Household and employment**				
**Is married/ in civil partnership**	118	20 (17%)	11 (9%)	**0.02**
**Living with partner**	89	31 (35%)	18 (20%)	**0.02**
**Natural children in household**	110	20 (18%)	15 (14%)	0.27
**Living with parents or legal guardian**	119	47 (40%)	75 (63%)	**<0.001**
**Own/mortgage house (if not living with parents)**	42	20 (48%)	16 (38%)	0.22
**Can drive a car**	111	86 (77%)	76 (68%)	**0.002**
**Income sources (more than one may apply)**				
Earnings	113	89 (79%)	76 (67%)	**0.02**
Pensions	113	9 (8%)	10 (9%)	1
Benefits	113	34 (30%)	42 (37%)	0.17
Credits	113	24 (21%)	16 (14%)	0.08
Allowances	113	6 (5%)	10 (9%)	0.42
No income	113	0 (0%)	4 (4%)	0.13
**Household receives Personal Independence Payment/Disability Allowance**	111	28 (25%)	22 (20%)	0.18
**Job status**				
Full time education	113	9 (8%)	25 (22%)	
Employed	113	72 (64%)	52 (46%)	
Unemployed	113	6 (5%)	4 (4%)	**0.02**
Unable to work due to health	113	23 (20%)	28 (25%)	
Homemaker	113	3 (3%)	4 (4%)	
**Working (vs not working)**	113	84 (74%)	81 (72%)	0.66
**Job type (among those working)**				
Elementary occupations	68	4 (6%)	12 (18%)	
Managers, directors and senior officials	83	6 (9%)	6 (9%)	
Professional occupations	83	13 (19%)	10 (15%)	
Associate professional and technical occupations	83	12 (18%)	7 (10%)	
Administrative and secretarial occupations	83	12 (18%)	13 (19%)	0.58
Skilled trades occupations	83	1 (1%)	1 (1%)	
Caring, leisure and other service occupations	83	11 (16%)	8 (12%)	
Sales and customer service occupations	83	8 (12%)	9 (13%)	
Process, plant and machine operatives	83	1 (1%)	2 (3%)	
**Works full-time**	90	64 (71%)	59 (66%)	0.36
**Degree/higher level education**	114	65 (57%)	50 (44%)	**0.002**

Total n=129. Percentages may not total 100 due to rounding. IMD, Index of multiple deprivation.

Table S3: change in additional self-reported psychological health outcomes between SPEAK-1 and SPEAK-2

Psychological characteristics	n	Possible range	SPEAK-2 median (IQR)	SPEAK-1 median (IQR)	*P*
**Body Image Scale**	107	0 - 30	9 (7 – 13)	10 (4 - 16)	0.48
**Social Impact Scale**	91	21 - 96	42 (31 – 54)	40 (29 - 56)	0.32
**Multidimensional Scale of Perceived Social Support**	107	12 - 84	69 (56 – 77)	67 (56 - 77)	0.15
**Patient Satisfaction Questionnaire-18 (PSQ-18)**					
General Satisfaction	106	1 - 5	4 (3.5 – 5)	4 (3 – 4.5)	0.11
Technical quality	106	1 - 5	4.25 (3.75 – 4.75)	4 (3.5 – 4.75)	**0.02**
Interpersonal manner	107	1 - 5	4.5 (4 – 5)	4 (4 – 5)	0.27
Communication	107	1 - 5	4 (3.5 – 4.5)	4 (3.5 – 5)	0.42
Financial aspects	106	1 - 5	4.5 (4.5 – 5)	4.5 (4 – 5)	**<0.001**
Time spent with doctor	107	1 - 5	4 (3 – 4.5)	4 (3 – 4.5)	0.26
Accessibility and convenience	105	1 - 5	4 (3.5 – 4.5)	3.75 (3.25 – 4.25)	0.06
**Psychological characteristics**	**n**	**Possible range**	**mean (SD)**	**mean (SD)**	***P***
**Multidimensional Health Locus of Control**					
Internal	98	6 - 36	17.68 (4.77)	22.71 (4.65)	**<0.001**
Chance	98	6 - 36	19.22 (4.60)	20.62 (5.35)	0.07
Powerful Others	98	6 - 36	18.19 (4.55)	22.10 (4.31)	**<0.001**
**Patient Activation Measure-13 (PAM-13)**	95	0 - 100	69.42 (16.03)	61.40 (15.58)	**<0.001**
**Acceptance of Illness Scale**	105	8 - 40	27.75 (7.44)	26.78 (7.13)	0.16

Total n=129. Nonparametric data are presented as median and IQR. Parametric data are presented as median and IQR. IQR, interquartile range; SD, standard deviation.
